# Angular Rate Estimation Using a Distributed Set of Accelerometers

**DOI:** 10.3390/s111110444

**Published:** 2011-11-02

**Authors:** Sungsu Park, Sung Kyung Hong

**Affiliations:** Department of Aerospace Engineering, Sejong University, Seoul 143-747, Korea; E-Mail: skhong@sejong.ac.kr

**Keywords:** distributed accelerometers, inertial measurement unit, Kalman filter, observability

## Abstract

A distributed set of accelerometers based on the minimum number of 12 accelerometers allows for computation of the magnitude of angular rate without using the integration operation. However, it is not easy to extract the magnitude of angular rate in the presence of the accelerometer noises, and even worse, it is difficult to determine the direction of a rotation because the angular rate is present in its quadratic form within the inertial measurement system equations. In this paper, an extended Kalman filter scheme to correctly estimate both the direction and magnitude of the angular rate through fusion of the angular acceleration and quadratic form of the angular rate is proposed. We also provide observability analysis for the general distributed accelerometers-based inertial measurement unit, and show that the angular rate can be correctly estimated by general nonlinear state estimators such as an extended Kalman filter, except under certain extreme conditions.

## 1. Introduction

A distributed accelerometers-based inertial measurement unit (IMU) uses only accelerometers to compute a specific force and angular rate. It has several advantages over the conventional gyroscope-equipped IMU, including robustness, easy calibration, low cost, and less power consumption. Furthermore, it can also function as an angular acceleration sensor, and thus it has applications in highly dynamic systems area, as well as low-cost, medium performance inertial navigation systems.

Development efforts for a distributed accelerometers-based IMU have been going on for over 30 years [1]. The research has mainly focused on the design of an optimal accelerometer configuration capable of overcoming the inherent calculation error that increases with time [2]. Although it was known in theory that a minimum of six accelerometers are required for a complete description of a rigid body motion, such six accelerometer schemes were not realized until Chen [3] proposed a cube-type IMU, which has one accelerometer at the center of each surface of a cube and its sensing direction is along the respective surface diagonal. This system was carefully evaluated and it was proved by Tan [4] that any six accelerometer configuration can be converted to the cube-type IMU with same computational simplicity. However, a six accelerometer-based IMU requires extra integration to obtain the angular rate, and thus a distributed accelerometers based inertial navigation system will have angular rate estimates that rapidly diverge, where the divergence rate is an order of magnitude greater than that of a gyroscope-equipped system. Park [5] proposed a redundant accelerometer-aided IMU with nine accelerometers by adding an extra three-axis accelerometer to the cube-type IMU to remove the extra integration using an extended Kalman filter, but his scheme suffers from an observability problem if one of the angular rate terms is zero.

The minimum number of accelerometers necessary to directly calculate the angular rate for a rigid body motion is 12 [6]. The accelerometer-based IMU with a minimum number of 12 accelerometers allows one to compute the angular rate without using the process of integration of the output of the accelerometers, and thus it can significantly improve the performance of the IMU. This scheme was studied further by Parsa [7] and Lin [8], and experimentally validated by Cappa [9]. However, the main problem with the 12 accelerometer-based IMU is that the angular rate is present in the system equations in its quadratic form, and therefore, it is difficult to extract the magnitude of the angular rate in the presence of accelerometer noises, and even worse, it is not possible to determine the direction of the angular rotation using only the quadratic form of the angular rate. In the literature, various methods have been proposed to solve this problem. Parsa [7] used a nonlinear least-square optimization procedure to estimate the angular rate from six measured quadratic forms of the angular rate. Cardou [10] reviewed various schemes available in the literature, and proposed a new algorithm for the estimation of the angular rate from the centripetal components of the acceleration measurements, while the sign of the angular rate was simply chosen by comparing the estimate with that of the integration of angular acceleration. Their schemes focused on extracting only the magnitude of angular rate in the presence of accelerometer noises. Continuous research efforts have been carried out to fuse the angular acceleration and the quadratic form of the angular rate. Schopp [11] applied an unscented Kalman filter to the entire 12 system equations to determine all the kinematic states such as specific force, angular rate and angular acceleration at the same time, but the observability was not proven. Lu [12] developed a new algorithm which derives the angular rate by mixing the signals from its quadratic form and its derivative form via context-based interacting multiple models, but his scheme only utilized the first three of the six quadratic angular rate terms, and the adequate context was set heuristically.

In contrast to those solutions, we propose in this paper an extended Kalman filter scheme to aid the integration of angular acceleration by six quadratic terms of angular rate in order to correctly estimate both the direction and magnitude of the angular rate. Compared to the previous works, we apply a Kalman filter to estimate angular rate only, since the specific force and angular acceleration can be computed algebraically. For an extended Kalman filter setup, the 4th–6th variables among the computed 12 kinematic variables in the system equation are designated as a state propagation equation, and the last six variables are designated as a measurement equation, which is the approach originally introduced in [5] by the authors. Furthermore, in this paper, we pay special attention on exploring the fundamental limit of the general distributed set of accelerometers based IMU using observability analysis, and formally show that the angular rate can be correctly estimated by general nonlinear state estimators such as the extended Kalman filter, except under certain extreme conditions.

## 2. Kinematics of the Distributed Accelerometers Based IMU

**Figure 1.**
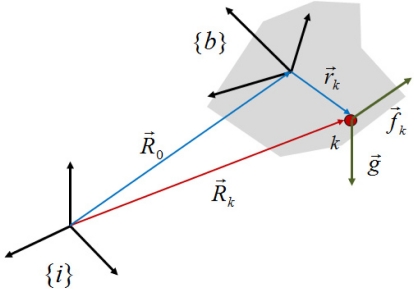
Inertial frame and body frame.

Consider the inertial frame {*i*} and a point *k* fixed in a rigid body moving in space, to which a body frame {*b*} is attached, as shown in Figure 1. $\vec{R_{0}}$ is the position vector from the center of the inertial frame to the center of the body frame, $\vec{R_{k}}$ is the position vector from the center of the inertial frame to a point *k*, and $\vec{r_{k}}$ is the position vector from the center of the body frame to the point *k*. Then, the acceleration of the point *k* with respect to the inertial frame is given by:

(1) $$\begin{array}{l} {\ddot{R}}_{k}^{i}={\ddot{R}}_{0}^{i}+C_{b}^{i}\left[{\dot{\omega }}_{\mathrm{ib}}^{b}\times \right]r_{k}^{b}+C_{b}^{i}{\left[\omega_{\mathrm{ib}}^{b}\times \right]}^{2}r_{k}^{b}\\ {\ddot{R}}_{k}^{i}=f_{k}^{i}+g^{i} \end{array}$$

where $f_{k}^{i}$ is the specific force at the point *k* and *g^i^* is the gravitational acceleration, and both are represented in the inertial frame {*i*}. Vector $\vec{r_{k}}$ is represented by $r_{k}^{b}$ in the body frame {*b*}, and $C_{b}^{i}$ is a direction cosine matrix that takes frame {*i*} to frame {*b*}. The term $\omega_{\mathrm{ib}}^{b}$ is the angular rate of frame {*b*} with respect to the frame {*i*}, represented in frame {*b*}, and $\left[\omega_{\mathrm{ib}}^{b}\times \right]$ is a cross-product matrix of the angular rate $\omega_{\mathrm{ib}}^{b}={\left[\begin{array}{ccc} \omega_{1} & \omega_{2} & \omega_{3} \end{array}\right]}^{T}$, which is given by:

$$[\omega_{\mathrm{ib}}^{b}\times ]=\left[\begin{array}{ccc} 0 & -\omega_{3} & \omega_{2}\\ \omega_{3} & 0 & -\omega_{1}\\ -\omega_{2} & \omega_{1} & 0 \end{array}\right]$$

If an accelerometer is rigidly attached at point *k* with the sensing direction $s_{k}^{b}$, the output *a_k_* of the accelerometer is given by:

(2) $$\begin{array}{lll} a_{k}(r_{k}^{b},s_{k}^{b}) & = & {\left(s_{k}^{b}\right)}^{T}f_{k}^{b}={\left(s_{k}^{b}\right)}^{T}C_{i}^{b}f_{k}^{i}\\ & = & {\left(s_{k}^{b}\right)}^{T}C_{i}^{b}({\ddot{R}}_{k}^{i}-g^{i})\\ & = & {\left(s_{k}^{b}\right)}^{T}f_{0}^{b}+{\left(s_{k}^{b}\right)}^{T}[{\dot{\omega }}_{\mathrm{ib}}^{b}\times ]r_{k}^{b}+{\left(s_{k}^{b}\right)}^{T}{\left[\omega_{\mathrm{ib}}^{b}\times \right]}^{2}r_{k}^{b} \end{array}$$

The output of the accelerometer is directly related with the specific force at the center of the body frame {*b*}, $f_{0}^{b}$, the rigid body angular acceleration ${\dot{\omega }}_{\mathrm{ib}}^{b}$, whose components appear in the skew-symmetric elements of $\left[{\dot{\omega }}_{\mathrm{ib}}^{b}\times \right]$ as follows:

(3) $$[{\dot{\omega }}_{\mathrm{ib}}^{b}\times ]=\left[\begin{array}{ccc} 0 & -{\dot{\omega }}_{3} & {\dot{\omega }}_{2}\\ {\dot{\omega }}_{3} & 0 & -{\dot{\omega }}_{1}\\ -{\dot{\omega }}_{2} & {\dot{\omega }}_{1} & 0 \end{array}\right]$$

and the angular rate $\omega_{\mathrm{ib}}^{b}$, whose components appear as quadratic products in the elements of ${\left[\omega_{\mathrm{ib}}^{b}\times \right]}^{2}$ as follows:

(4) $${\left[\omega_{\mathrm{ib}}^{b}\times \right]}^{2}=\left[\begin{array}{ccc} -(\omega_{2}^{2}+\omega_{3}^{2}) & \omega_{1}\omega_{2} & \omega_{1}\omega_{3}\\ \omega_{1}\omega_{2} & -(\omega_{1}^{2}+\omega_{3}^{2}) & \omega_{2}\omega_{3}\\ \omega_{1}\omega_{3} & \omega_{2}\omega_{3} & -(\omega_{1}^{2}+\omega_{2}^{2}) \end{array}\right]$$

Through simple algebraic manipulations, it is easily shown that Equation (2) can be expressed with 12 kinematic variables as follows:

(5) $$a_{k}(r_{k}^{b},s_{k}^{b})=J_{k}y$$

(6) $$y={\left[\begin{array}{llllllllllll} f_{1} & f_{2} & f_{3} & {\dot{\omega }}_{1} & {\dot{\omega }}_{2} & {\dot{\omega }}_{3} & \omega_{1}^{2} & \omega_{2}^{2} & \omega_{3}^{2} & \omega_{1}\omega_{2} & \omega_{1}\omega_{3} & \omega_{2}\omega_{3} \end{array}\right]}^{T}$$

(7) $$\begin{array}{l} J_{k}=[\begin{array}{lllllll} s_{1} & s_{2} & s_{3} & -r_{3}s_{2}+r_{2}s_{3} & r_{3}s_{1}-r_{1}s_{3} & -r_{2}s_{1}+r_{1}s_{2} & \cdots \end{array}\\ \;\;\;\;\;\;\;\;\;\;\;\;\;\;\;\;\;\;\begin{array}{llllll} -r_{2}s_{2}-r_{3}s_{3} & -r_{1}s_{1}-r_{3}s_{3} & -r_{1}s_{1}-r_{2}s_{2} & r_{2}s_{1}+r_{1}s_{2} & r_{3}s_{1}+r_{1}s_{3} & r_{3}s_{2}+r_{2}s_{3} \end{array}] \end{array}$$

where in *f_i_*’s, *r_i_*’s, and *s_i_*’s (for *i* = 1, 2, 3) denote the components of $f_{0}^{b}$, $r_{k}^{b}$ and $s_{k}^{b}$, respectively.

Now suppose that *N* accelerometers are distributed in the body frame, and if we collect the accelerometer outputs in a vector form, we have:

(8) $$A=\left[\begin{array}{c} a_{1}\\ \cdots \\ a_{N} \end{array}\right]=\left[\begin{array}{c} J_{1}\\ \cdots \\ J_{N} \end{array}\right]y=Jy$$

where the *N* × 12 matrix *J* is called a configuration matrix, and it consists of the constant parameters about the positions and the sensing directions of the accelerometer array.

If the configuration matrix *J* has a left inverse matrix, then it is possible to calculate 12 kinematic variables as follows:

(9) $$y=J^{+}A$$

where *J*^+^ = (*J^T^J*)^−1^*J^T^* is left inverse matrix of *J*, for which to exist, a minimum of 12 accelerometers is necessary. Equation (9) is called the system equation.

**Figure 2.**
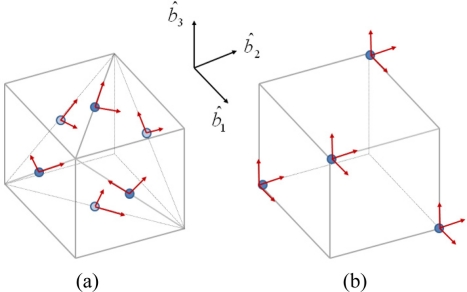
Two possible configurations (**a**) configuration A; (**b**) configuration B.

For a configuration of 12 accelerometers, the left inverse matrix *J*^+^ becomes the inverse matrix *J*^−1^, and the main requirement for the placement of the accelerometers is that the configuration matrix is invertible. Several accelerometer array configurations have been proposed in the literature, and Figure 2 shows two feasible configurations as examples.

Additional requirements can be imposed to determine the positions and the sensing directions of the accelerometers, since we have 60 parameters for an array of 12 accelerometers to be determined. For example, we can try to find an accelerometer array configuration yielding the minimum influence of the accelerometer noise on the variances of the kinematic variables, since it depends on the accelerometer configuration as follows:

(10) $$X_{y}=J^{-1}X_{A}J^{-T}=\sigma_{a}^{2}{\left(J^{T}J\right)}^{-1}$$

where *X_y_* and *X_A_* are the covariance matrices of the kinematic variables and the accelerometer array, respectively, and $\sigma_{a}^{2}$ is the variance of the single accelerometer. The second term of Equation (10) is valid under the assumption that the accelerometer array is i.i.d. For another example, Lin [8] studied the accelerometer array configuration producing a minimum condition number of *J*, and pointed out that placement of the four 3-axis accelerometers shown in Figure 2(b) yields the best condition number of *J*, which indicates that this configuration is the most appropriate for matrix inversion, although the inversion of the configuration matrix *J* can be computed offline only once.

## 3. Estimation of the Angular Rate

The 12 kinematic variables can be computed from Equation (9), and the specific force at the center of the body frame, angular acceleration, and the magnitude of the angular rate can be obtained. Therefore, a distributed accelerometer-based IMU can perform the same function as a conventional gyro-equipped IMU, except for the direction of the angular rotation, whose magnitude is directly measured by gyroscopes in a conventional gyro-equipped IMU. It is not straightforward to determine the sign of the angular rate using a distributed accelerometer-based IMU since the angular rate is present in its quadratic form, as shown in Equation (6).

Several methods have been proposed to resolve this sign ambiguity. Some methods resort to additional sensors such as the conventional low-cost gyroscopes or GPS to determine the correct sign of the angular rate. However, the common approaches compare the estimates with those obtained from the integration of the angular acceleration. In this paper, we use an extended Kalman filter to fuse two sensed sources, the angular acceleration and the quadratic form of the angular rate, and thus to estimate the sign and magnitude of the angular rate at the same time.

For an extended Kalman filter setup, the angular accelerations, which are the 4th–6th terms of *y* in Equation (6), are designated to establish the angular rate propagation equation, and the quadratic forms of the angular rate, which are the last six terms of *y*, are designated to construct angular rate measurement equation.

Then, the angular rate propagation and measurement equations are written as:

(11) $${\dot{\omega }}_{\mathrm{ib}}^{b}=\alpha_{\mathrm{ib}}^{b}:\left[\begin{array}{c} {\dot{\omega }}_{1}\\ {\dot{\omega }}_{2}\\ {\dot{\omega }}_{3} \end{array}\right]=\left[\begin{array}{c} \alpha_{1}\\ \alpha_{2}\\ \alpha_{3} \end{array}\right]$$

(12) $$z=h(\omega_{\mathrm{ib}}^{b}):\left[\begin{array}{c} z_{1}\\ z_{2}\\ z_{3}\\ z_{4}\\ z_{5}\\ z_{6} \end{array}\right]=\left[\begin{array}{c} \omega_{1}^{2}\\ \omega_{2}^{2}\\ \omega_{3}^{2}\\ \omega_{1}\omega_{2}\\ \omega_{1}\omega_{3}\\ \omega_{2}\omega_{3} \end{array}\right]$$

where $\alpha_{\mathrm{ib}}^{b}={\left[\begin{array}{ccc} \alpha_{1} & \alpha_{2} & \alpha_{3} \end{array}\right]}^{T}$ are the computed angular acceleration from Equation (9).

### 3.1. Observability Analysis

Observability denotes the ability to uniquely determine the states of a dynamic system from its measurements and it is the indication for the feasibility of design of a state estimator such as a Kalman filter [13]. Observability can be examined by the rank of the corresponding observability matrix, and the observability matrix of nonlinear systems (11) and (12) is given as follows:

(13) $$W_{0}=\left[\begin{array}{l} \nabla z\\ \nabla \dot{z}\\ \nabla \ddot{z} \end{array}\right]$$

where *W*_0_ is a 18 × 3 matrix, and ∇ stands for a gradient operator with respect to $\omega_{\mathrm{ib}}^{b}$, which is given by:

$$\nabla z=\left[\begin{array}{ccc} 2\omega_{1} & 0 & 0\\ 0 & 2\omega_{2} & 0\\ 0 & 0 & 2\omega_{3}\\ \omega_{2} & \omega_{1} & 0\\ \omega_{3} & 0 & \omega_{1}\\ 0 & \omega_{3} & \omega_{2} \end{array}\right]$$

As seen from Equation (13), the observability property can be changed by the angular acceleration as well as the angular rate itself to be measured. There are three cases to be considered for the observability analysis.

In the first case, wherein at least one of angular acceleration terms is not zero, the rank of observability matrix is three and thus the system is observable for every angular rate including the origin of angular rate vector space, *i.e*., $\omega_{\mathrm{ib}}^{b}={\left[\begin{array}{ccc} 0 & 0 & 0 \end{array}\right]}^{T}$, which means that the system is globally observable in the entire angular rate vector space.

**Figure 3.**
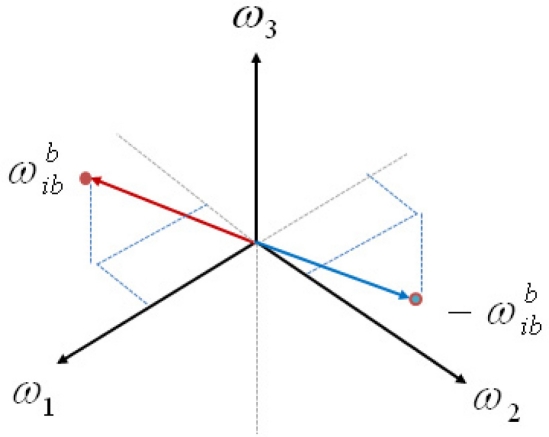
Two possible solutions in angular rate space.

In the second case, in which all of angular rate terms are constant, the system becomes static but it is still observable if at least one of angular rate terms is not zero since the rank of observability matrix is three. However, the observability condition fails at the origin of angular rate vector space, which means that the system is locally observable in the angular rate vector space. Indeed, it is not possible to determine the direction of angular rate using only quadratic terms of the angular rate, since the quadratic terms do not give a unique solution. Instead, it yields two possible solutions, each of which having equal magnitude but opposite direction in the angular rate vector space, as shown in Figure 3. Thus, if an *a priori* estimate of angular rate is closer to the true angular rate than to the false angular rate, then the estimate converges to the true value. However, if the *a priori* estimate of angular rate is closer to the false angular rate, then the estimate goes to the false value. Thus, if there is no information on the angular rate at a previous time, it cannot be guaranteed for the angular rate to be correctly estimated.

In the general application there are certain instants where the second case may occur. However, this is not a problem because the recursive predictor-corrector scheme of Kalman filter makes it possible to continuously incorporate an *a priori* estimate of angular rate, which must be close to the true angular rate, into the filtering process. Instead, the extremely severe situation is the case in which all of the angular rates are constant from the initial time when the system starts to work, because there is no available information for the initial guess on the direction of a rotation. Although this hardly occurs in real world applications, this situation may be avoided by flipping the sign of angular rate estimate where necessary when the divergence of the filter is detected.

The last case is a special case in which all of angular rate and angular acceleration terms are zero. Since all elements of observability matrix are zero, the system becomes completely unobservable. However, although the system is completely unobservable, this case can be treated easily since we can be informed that all angular accelerations and rates are zero from the designated measurement equation.

### 3.2. Extended Kalman Filter and Its Modification

The angular rate propagation Equation (11) and measurement Equation (12) are re-written here in discrete-time form:

(14) $$\begin{array}{l} \omega_{\mathrm{ib}}^{b}(t_{k+1})=\omega_{\mathrm{ib}}^{b}(t_{k})+\frac{\Delta T}{2}({\dot{\omega }}_{\mathrm{ib}}^{b}(t_{k})+{\dot{\omega }}_{\mathrm{ib}}^{b}(t_{k+1}))+w(t_{k})\\ z(t_{k})=h(\omega_{\mathrm{ib}}^{b}(t_{k}))+v(t_{k}) \end{array}$$

where the subscript *k* denotes the time update sequence and Δ*T* is the sampling interval. The terms *w*(*t_k_*) and *v*(*t_k_*) are included to account for noise in the computations of the angular acceleration and the quadratic forms of the angular rate with Equation (9). They originate from noises in the accelerometer outputs, which are assumed to be uncorrelated zero-mean white with the known power spectral densities. The covariance matrices *Q* and *R* of *w*(*t_k_*) and *v*(*t_k_*) can be computed using Equation (10).

An extended Kalman filter can be designed using these two equations. The time update of the angular rate estimate and its covariance matrix are given by:

(15) $$\begin{array}{l} {\hat{\omega }}_{\mathrm{ib}}^{b}(t_{k+1}|t_{k})={\hat{\omega }}_{\mathrm{ib}}^{b}(t_{k}|t_{k})+\frac{\Delta T}{2}({\dot{\omega }}_{\mathrm{ib}}^{b}(t_{k})+{\dot{\omega }}_{\mathrm{ib}}^{b}(t_{k+1}))\\ P(t_{k+1}|t_{k})=P(t_{k}|t_{k})+Q \end{array}$$

where ${\hat{\omega }}_{\mathrm{ib}}^{b}(t_{k+1}|t_{k})$ and *P*(*t_k_*_+1_|*t_k_*) are the *a priori* estimates of angular rate and error covariance. The time update portion of the extended Kalman filter gives a prediction of the angular rate at time *t_k_*_+1_, along with the associated error covariance *P*. The extended Kalman filter gain, angular rate estimate and its covariance are updated as follows [14]:

(16) $$\begin{array}{l} K(t_{k+1})=P(t_{k+1}|t_{k})H^{T}(t_{k+1}){\left(H(t_{k+1})P(t_{K+1}|t_{k})H^{T}(t_{k+1})+R\right)}^{-1}\\ {\hat{\omega }}_{\mathrm{ib}}^{b}(t_{k+1}|t_{k+1})={\hat{\omega }}_{\mathrm{ib}}^{b}(t_{k+1}|t_{k})+K(t_{K+1})[z(t_{k+1})-\hat{z}(t_{k+1})]\\ P(t_{k+1}|t_{k+1})=(I-K(t_{k+1})H(t_{k+1}))P(t_{k+1}|t_{k}) \end{array}$$

where:

(17) $$\begin{array}{l} H(t_{k+1})={\left[\frac{\partial h(\omega_{\mathrm{ib}}^{b})}{\partial \omega_{\mathrm{ib}}^{b}}\right]}_{\omega_{\mathrm{ib}}^{b}={\hat{\omega }}_{\mathrm{ib}}^{b}(t_{k+1}|t_{k})}\\ \hat{z}(t_{k+1})=h({\hat{\omega }}_{\mathrm{ib}}^{b}(t_{k+1}|t_{k})) \end{array}$$

The measurement update provides a correction based on the measurement *z* at time *t_k_*_+1_ to yield the *a posteriori* estimate and its covariance. For the case in which all of both angular acceleration and quadratic angular rate terms lie under the certain thresholds and can be considered as zero, the measurement update portion can be modified as follows:

(18) $$\begin{array}{l} {\hat{\omega }}_{\mathrm{ib}}^{b}(t_{k+1}|t_{k+1})=0\\ P(t_{k+1}|t_{k+1})=P(t_{k+1}|t_{k}) \end{array}$$

The thresholds for angular acceleration and quadratic angular rate terms can be chosen by several methods. One method may be using the standard deviation of their measurement noises, and another method may be using a moving average of the measurements to obtain smoother transition.

## 4. Simulation Results

To evaluate the performance of the proposed scheme for the angular rate estimation, computer simulations are performed. We use the 12 accelerometer-based IMU shown in Figure 2(b). The IMU cube length *l* is 10 cm, and the position of the accelerometer array is determined as follows:

(19) $$r^{b}=l\cdot \left[\begin{array}{cccccccccccc} -1 & -1 & -1 & 1 & 1 & 1 & -1 & -1 & -1 & 1 & 1 & 1\\ 1 & 1 & 1 & -1 & -1 & -1 & -1 & -1 & -1 & 1 & 1 & 1\\ 1 & 1 & 1 & 1 & 1 & 1 & -1 & -1 & -1 & -1 & -1 & -1 \end{array}\right]$$

and the sensing direction is determined as follows:

(20) $$s^{b}=\left[\begin{array}{cccccccccccc} 1 & 0 & 0 & 1 & 0 & 0 & 1 & 0 & 0 & 1 & 0 & 0\\ 0 & 1 & 0 & 0 & 1 & 0 & 0 & 1 & 0 & 0 & 1 & 0\\ 0 & 0 & 1 & 0 & 0 & 1 & 0 & 0 & 1 & 0 & 0 & 1 \end{array}\right]$$

The noise incorporated in the simulation is white, and the noise associated with each accelerometer measurement is assumed to have the same density of 100 μg/ $\sqrt{\text{Hz}}$. The other errors such as scale factor error, bias, and the configuration (position and sensing direction) error of the accelerometer are assumed to be identified and compensated by some calibration scheme beforehand. The time update of the IMU algorithm is 100 Hz. Simulations are performed for three different cases as follows.

### 4.1. Case 1: Arbitrary Trajectory

**Figure 4.**
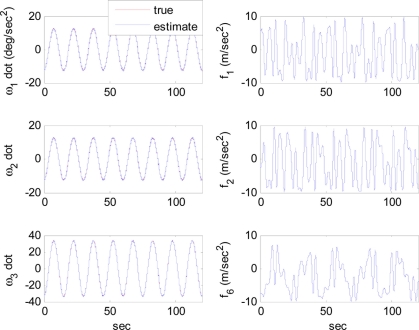
Reference trajectories of angular acceleration and specific force: Case 1.

**Figure 5.**
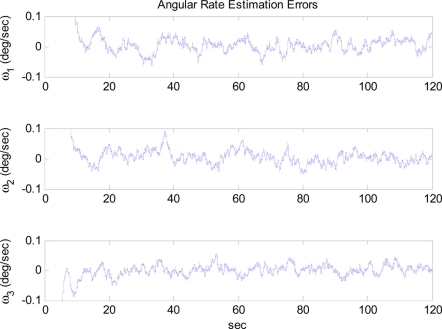
Angular rate estimation errors: Case 1.

The arbitrary trajectory shown in Figure 4 is used for the simulations. Figure 4 shows that the specific force and angular acceleration converge to their true values. Since the calculation of specific force and angular acceleration uses measurements obtained directly from the accelerometers, it never fails to identify them. In this simulation, the trajectory to be followed is sinusoidal. Thus, the condition of observability is satisfied and the extended Kalman filter is expected to estimate the angular rate correctly, as discussed previously. Figure 5 shows that the angular rate is correctly estimated by the extended Kalman filter.

### 4.2. Case 2: Initially at Rest followed by Time-Varying Angular Acceleration

**Figure 6.**
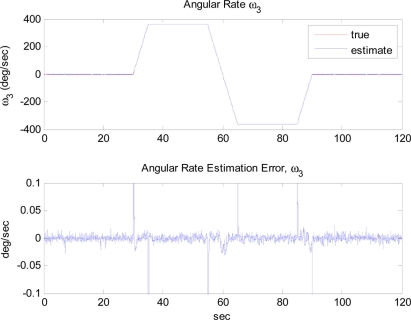
Angular rate estimation error of *ω*_3_: Case 2.

**Figure 7.**
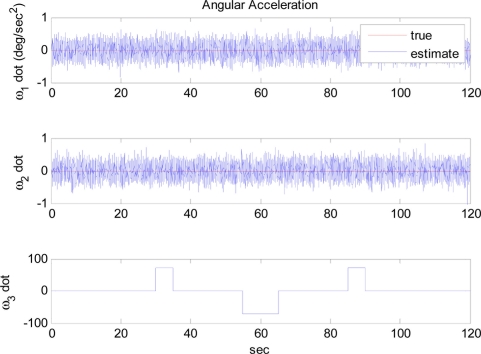
Angular acceleration: Case 2.

Figure 6 shows the trajectory of angular rate *ω*_3_, used for the simulations. Other angular rate and angular acceleration terms are assumed to be zero, as shown in Figure 7. The extended Kalman filter correctly estimates the angular rate including the direction of it, which is expected by the observability analysis. Note that no drift of the estimates occurs when no angular rate is present, thanks to the modified measurement update portion.

### 4.3. Case 3: Constant Angular Rate

The extremely severe situation for distributed an accelerometer-based IMU is the case in which all of the angular rates are constant from the initial time when IMU is on, because there is no available information on the direction of rotation. According to the observability analysis, the extended Kalman filter cannot guarantee that the angular rate estimate will converge toward the correct values, unless the initial angular rate estimate is closer to the true angular rate than to the false angular rate.

**Figure 8.**
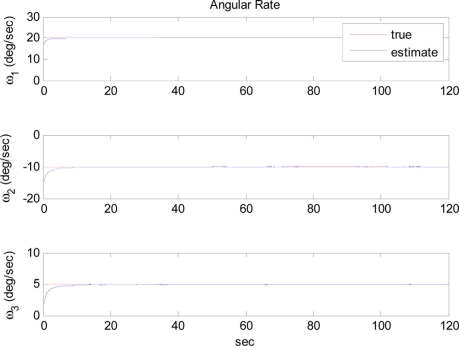
Angular rate estimation with close initial conditions: Case 3-1.

**Figure 9.**
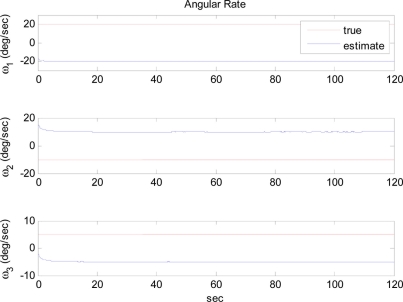
Angular rate estimation with far initial conditions: Case 3-1.

The simulation was performed with two different initial estimates within the extended Kalman filter. Figure 8 shows the simulation results when the initial angular rate estimate is closer to the true angular rate. As discussed in the observability analysis, the estimate converges to the true angular rate. Figure 9, on the other hand, shows that the estimate converges to the false angular rates, which has the same magnitude but opposite direction and thus produces same values of quadratic angular rate terms. The two results agree well with our analytic arguments.

**Figure 10.**
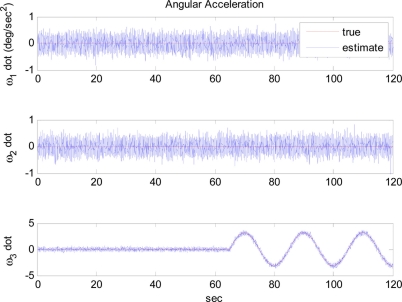
Angular acceleration: Case 3-2.

**Figure 11.**
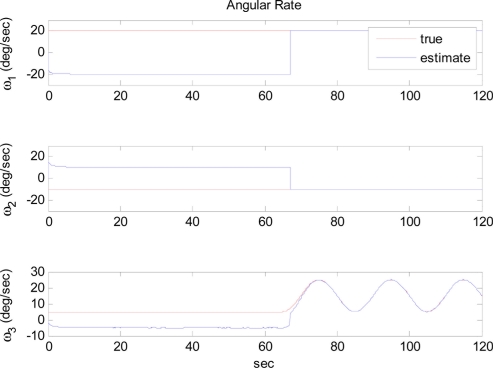
Angular rate estimation: Case 3-2.

**Figure 12.**
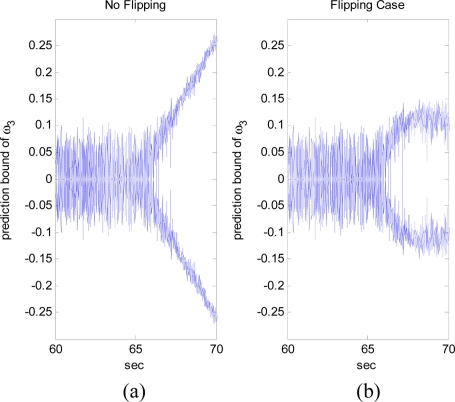
Response of measurement prediction covariance: (**a**) no flipping; (**b**) flipping cases.

The situation shown in Figure 9 can be avoided when the angular acceleration starts to vary as shown in Figure 10, where the trajectory of angular acceleration *ω̇*_3_ follows a sinusoidal pattern at 65 s. As shown in Figure 11, although initially the estimate converges to the false angular rate, eventually it converges to the true value. This is achieved by flipping the sign of angular rate estimate when the divergence of the filter is detected around at 67 s. In this simulation, the divergence of the filter is detected based on the magnitude of the measurement prediction covariance of the filter as shown in Figure 12. The measurement prediction covariance, *S*(*t_k_*), is given by following equation [14]:

(21) $$S(t_{k})=H(t_{k})P(t_{k}|t_{k-1})H^{T}(t_{k})+R$$

## 5. Conclusions

Although a typical 12 distributed accelerometer-based IMU allows for the computing of the magnitude of an angular rate without using the integration of the outputs of accelerometers, it is difficult to determine the direction of a rotation because the angular rate is present in its quadratic form within the IMU system equations. To tackle this inherent disadvantage, we proposed an extended Kalman filter scheme to aid the integration of angular acceleration by six quadratic terms of angular rate in order to correctly estimate both the direction and magnitude of the angular rate. We also provided observability analysis for the general 12 accelerometer-based IMU, and showed that the angular rate can be correctly estimated by general nonlinear state estimators such as an extended Kalman filter, except under certain extreme conditions.

The performance of the proposed scheme for the angular rate estimation was evaluated using computer simulations under three special cases. The simulation results agree well with our analyses, and show that the proposed scheme correctly estimates specific force, angular acceleration, and the direction and magnitude of the angular rate as well.
